# Supplementary material for: A Likelihood Approach for Real-Time Calibration of Stochastic Compartmental Epidemic Models
Source: PLoS Comput Biol. 2017 Jan 17;13(1):e1005257. doi: 10.1371/journal.pcbi.1005257 (PMC5240920; doi:10.1371/journal.pcbi.1005257)
Supplement: S1 File — (TAR.GZ) [file pcbi.1005257.s014.tar.gz › HSPH_Online-SI-Revision/IPoi09/n100-extreme/n100-extreme_BENCH_table0.pdf]

1	R0	{2.76052, 16.3913}	{2.96947, 15.963}	{{2.96947, 15.963}, {2.96947, 15.963}}	{7.56923, 2.61297}	inf	26580.	24785.9	{19741., 29422.}	13.7125	0.0795545	newly	10855.	10271.4	{7855., 12460.}	14.4763	0.0758911	Reff	0.831027	0.869326	{0.442065, 1.74599}	42.7327	ar	92090.	93829.	{93704., 93974.}	1.88835
2	R0	{2.18202, 15.1734}	{2.93978, 19.9817}	{{2.93978, 19.9817}, {2.93978, 19.9817}}	{34.7275, 31.689}	inf	17999.	17255.9	{11429., 24300.}	23.4258	0.108543	newly	8027.	5572.89	{3524., 8114.}	34.7199	0.198599	Reff	0.873157	0.476888	{0.287716, 0.732946}	57.804	ar	84264.	93627.3	{93451., 93834.}	11.1118
3	R0	{2.57891, 12.5971}	{2.86001, 13.5542}	{{2.86001, 13.5542}, {2.86001, 13.5542}}	{10.9, 7.59778}	inf	22711.	18887.2	{12532., 26034.}	23.1934	0.121321	newly	11421.	8733.67	{5501., 12891.}	29.1168	0.160024	Reff	0.727149	0.555074	{0.320922, 0.785874}	45.58	ar	90302.	92998.7	{92780., 93186.}	2.98628
4	R0	{2.5856, 9.28508}	{2.99092, 10.3743}	{{2.99092, 10.3743}, {2.99092, 10.3743}}	{15.6761, 11.7309}	inf	23144.	24478.5	{19394., 29490.}	17.4446	0.0784227	newly	15345.	14644.8	{10884., 18757.}	18.2086	0.0856603	Reff	0.760942	0.728326	{0.411162, 1.19738}	38.9717	ar	90312.	93982.4	{93850., 94113.}	4.06417
5	R0	{2.4406, 12.1341}	{2.86001, 13.5542}	{{2.86001, 13.5542}, {2.86001, 13.5542}}	{17.1847, 11.7034}	inf	22895.	22403.5	{16365., 27756.}	16.194	0.0772647	newly	12782.	10696.5	{7205., 13973.}	19.8146	0.10373	Reff	0.972189	0.760428	{0.401889, 1.19034}	45.255	ar	88597.	92998.7	{92780., 93186.}	4.9682
6	R0	{2.72155, 15.4094}	{2.96947, 15.963}	{{2.96947, 15.963}, {2.96947, 15.963}}	{9.10951, 3.59261}	inf	26147.	18275.9	{11765., 25054.}	31.8971	0.179243	newly	11271.	7212.66	{4444., 10457.}	38.1068	0.22295	Reff	0.880857	0.496193	{0.288811, 0.847576}	57.1709	ar	91682.	93829.	{93704., 93974.}	2.34177
7	R0	{2.63781, 14.3935}	{2.96978, 18.8857}	{{2.96978, 18.8857}, {2.96978, 18.8857}}	{12.5851, 31.2099}	inf	24939.	25081.1	{16193., 29530.}	18.8056	0.111964	newly	11237.	9197.35	{7326., 10857.}	18.1512	0.112805	Reff	0.790499	1.14561	{0.555705, 2.1851}	59.1235	ar	91192.	93825.4	{93652., 93970.}	2.88776
8	R0	{2.82391, 16.104}	{2.96947, 15.963}	{{2.96947, 15.963}, {2.96947, 15.963}}	{5.15456, 0.875559}	inf	27368.	21902.1	{15562., 27593.}	20.9514	0.11402	newly	11207.	8804.21	{6090., 11525.}	23.5482	0.126884	Reff	0.861801	0.649952	{0.346834, 1.23952}	50.2519	ar	92664.	93829.	{93704., 93974.}	1.25721
9	R0	{2.80645, 14.6457}	{2.76203, 15.0558}	{{2.76203, 15.0558}, {2.76203, 15.0558}}	{1.58278, 2.80014}	inf	27606.	22800.3	{13799., 26705.}	17.4082	0.105801	newly	12694.	10420.8	{6913., 12413.}	17.9259	0.100616	Reff	0.94945	1.08368	{0.55097, 1.97339}	43.6134	ar	92567.	92152.	{91982., 92321.}	0.448306
10	R0	{1.86213, 5.4271}	{2.93653, 10.872}	{{2.93653, 10.872}, {2.93653, 10.872}}	{57.6974, 100.328}	inf	10896.	22491.1	{16377., 28242.}	106.417	0.305458	newly	11765.	12848.9	{9096., 17367.}	22.6242	0.0914238	Reff	0.763157	0.674098	{0.380369, 1.23513}	42.6551	ar	75362.	93586.3	{93426., 93765.}	24.1824
11	R0	{2.27349, 14.6338}	{2.8833, 17.1847}	{{2.8833, 17.1847}, {2.8833, 17.1847}}	{26.8226, 17.4316}	inf	19721.	21138.9	{14411., 27811.}	20.5257	0.0865423	newly	8893.	7970.84	{5501., 10428.}	19.952	0.0985773	Reff	0.854264	0.654181	{0.355107, 1.18841}	47.6007	ar	85805.	93197.4	{93009., 93386.}	8.61532
12	R0	{2.77261, 14.1894}	{2.8833, 17.1847}	{{2.8833, 17.1847}, {2.8833, 17.1847}}	{3.99227, 21.1094}	inf	27138.	24646.6	{16842., 28451.}	12.6214	0.0837424	newly	12745.	9895.8	{8117., 11584.}	22.3554	0.128921	Reff	0.865526	1.10214	{0.561465, 2.0688}	46.7889	ar	92508.	93197.4	{93009., 93386.}	0.745208
13	R0	{2.65402, 19.3935}	{2.93978, 19.9817}	{{2.93978, 19.9817}, {2.93978, 19.9817}}	{10.7671, 3.03297}	inf	25765.	22678.7	{17537., 28417.}	17.1666	0.0900498	newly	8948.	7502.05	{5522., 9707.}	19.5866	0.102676	Reff	0.987375	0.730324	{0.391873, 1.31658}	49.8555	ar	91184.	93627.3	{93451., 93834.}	2.6795
14	R0	{1.75297, 4.45354}	{2.99911, 15.6207}	{{2.99911, 15.6207}, {2.99911, 15.6207}}	{71.0874, 250.748}	inf	10727.	24686.2	{11047., 29903.}	141.231	0.396303	newly	15310.	11390.3	{6787., 13327.}	25.6021	0.163535	Reff	0.926059	1.42073	{0.732353, 2.4752}	62.5761	ar	71263.	94013.6	{93846., 94150.}	31.9249
15	R0	{1.99928, 7.1501}	{2.99939, 13.7076}	{{2.99939, 13.7076}, {2.99939, 13.7076}}	{50.0235, 91.712}	inf	13996.	25166.	{20084., 29359.}	82.1777	0.259209	newly	12056.	11899.3	{9024., 14406.}	14.3336	0.0667747	Reff	0.80563	0.784675	{0.428583, 1.39244}	39.9475	ar	79602.	94036.2	{93909., 94200.}	18.133
16	R0	{2.67398, 10.3134}	{2.99939, 13.7076}	{{2.99939, 13.7076}, {2.99939, 13.7076}}	{12.1695, 32.9106}	inf	23578.	21128.	{15199., 28201.}	18.2237	0.0893992	newly	14011.	9586.95	{6589., 13558.}	32.4403	0.182681	Reff	0.705449	0.548882	{0.326004, 0.88359}	44.6324	ar	91289.	94036.2	{93909., 94200.}	3.00939
17	R0	{2.44634, 16.3226}	{2.97157, 19.3547}	{{2.97157, 19.3547}, {2.97157, 19.3547}}	{21.47, 18.5761}	inf	22666.	24874.	{19615., 29545.}	22.1481	0.10642	newly	9419.	8722.1	{6588., 10513.}	14.5958	0.0798665	Reff	0.907103	0.97577	{0.470875, 1.86897}	47.1678	ar	88710.	93849.1	{93703., 93993.}	5.79312
18	R0	{2.02168, 10.1676}	{2.97157, 19.3547}	{{2.97157, 19.3547}, {2.97157, 19.3547}}	{46.9852, 90.3566}	inf	15535.	25013.6	{14529., 29699.}	72.9503	0.262556	newly	10392.	9066.24	{6436., 10612.}	13.8626	0.0941453	Reff	0.996001	1.24103	{0.607954, 2.27456}	47.3748	ar	80299.	93849.1	{93703., 93993.}	16.8745
19	R0	{2.89969, 15.009}	{2.76203, 15.0558}	{{2.76203, 15.0558}, {2.76203, 15.0558}}	{4.7474, 0.311813}	inf	28996.	22800.3	{13799., 26705.}	21.3675	0.127136	newly	12862.	10420.8	{6913., 12413.}	18.9798	0.106247	Reff	0.881738	1.08368	{0.55097, 1.97339}	46.6201	ar	93349.	92152.	{91982., 92321.}	1.28227
20	R0	{2.68933, 7.34928}	{2.93653, 10.872}	{{2.93653, 10.872}, {2.93653, 10.872}}	{9.19188, 47.9329}	inf	24920.	25316.4	{19236., 29202.}	14.8783	0.0781279	newly	20039.	15638.3	{11973., 18530.}	21.9608	0.117337	Reff	0.765733	1.05036	{0.56907, 1.97379}	52.3759	ar	91574.	93586.3	{93426., 93765.}	2.19747
21	R0	{2.15352, 9.28074}	{2.95209, 14.037}	{{2.95209, 14.037}, {2.95209, 14.037}}	{37.0821, 51.2487}	inf	17876.	25378.8	{20204., 29275.}	48.5999	0.177641	newly	12361.	12100.6	{9553., 14419.}	12.9374	0.061656	Reff	0.8829	0.976461	{0.535775, 1.9335}	42.13	ar	83845.	93710.9	{93555., 93857.}	11.7668
22	R0	{2.21953, 9.04387}	{2.9884, 12.8326}	{{2.9884, 12.8326}, {2.9884, 12.8326}}	{34.6411, 41.8928}	inf	18399.	25608.5	{20203., 29739.}	43.2585	0.159782	newly	13126.	12970.3	{9877., 15724.}	14.5102	0.0683359	Reff	0.851523	0.842733	{0.473303, 1.45882}	39.1927	ar	84856.	93983.5	{93851., 94123.}	10.7565
23	R0	{2.21987, 14.5347}	{2.93978, 19.9817}	{{2.93978, 19.9817}, {2.93978, 19.9817}}	{32.4303, 37.4758}	inf	18816.	22678.7	{17537., 28417.}	27.7173	0.108325	newly	8717.	7502.05	{5522., 9707.}	18.8289	0.0972072	Reff	0.868968	0.730324	{0.391873, 1.31658}	47.0635	ar	85067.	93627.3	{93451., 93834.}	10.063
24	R0	{2.84268, 17.9659}	{2.7811, 19.6712}	{{2.7811, 19.6712}, {2.7811, 19.6712}}	{2.16627, 9.49187}	inf	27580.	23309.3	{17627., 27162.}	15.4966	0.0957802	newly	10377.	7992.23	{5594., 9567.}	22.9813	0.13126	Reff	0.920602	0.912475	{0.526323, 1.45018}	37.6424	ar	92836.	92343.4	{92163., 92489.}	0.530631
25	R0	{2.52706, 10.3679}	{2.8833, 17.1847}	{{2.8833, 17.1847}, {2.8833, 17.1847}}	{14.097, 65.7491}	inf	22248.	24646.6	{16842., 28451.}	24.3589	0.119737	newly	13481.	9895.8	{8117., 11584.}	26.5944	0.153303	Reff	0.761075	1.10214	{0.561465, 2.0688}	56.3038	ar	89623.	93197.4	{93009., 93386.}	3.98824
26	R0	{2.62835, 19.1088}	{2.97157, 19.3547}	{{2.97157, 19.3547}, {2.97157, 19.3547}}	{13.0584, 1.28684}	inf	24689.	23253.	{17249., 29106.}	16.2876	0.0824841	newly	8698.	7976.1	{5517., 10469.}	17.9085	0.0892647	Reff	0.928833	0.764755	{0.380836, 1.43227}	48.9254	ar	90908.	93849.1	{93703., 93993.}	3.23523
27	R0	{2.76831, 19.4155}	{2.96398, 17.7348}	{{2.96398, 17.7348}, {2.96398, 17.7348}}	{7.06821, 8.65649}	inf	25708.	18504.3	{11730., 27462.}	30.8301	0.173175	newly	8776.	6648.71	{4156., 10299.}	31.4229	0.170917	Reff	0.75287	0.491713	{0.288069, 0.825794}	50.667	ar	92175.	93780.3	{93638., 93879.}	1.74153
28	R0	{2.96609, 16.8903}	{2.79531, 12.1249}	{{2.79531, 12.1249}, {2.79531, 12.1249}}	{5.75775, 28.2138}	inf	28896.	17300.1	{11417., 23758.}	40.1297	0.237811	newly	11093.	8706.3	{5337., 12524.}	29.1965	0.155758	Reff	0.743925	0.499221	{0.32448, 0.649099}	46.1021	ar	94024.	92457.4	{92317., 92603.}	1.66622
29	R0	{2.17046, 14.39}	{2.85855, 19.9832}	{{2.86095, 19.9929}, {2.86095, 19.9929}}	{31.7027, 38.869}	inf	18683.	24939.7	{21454., 28222.}	41.0549	0.166936	newly	8778.	8578.77	{6966., 9842.}	12.0306	0.0682615	Reff	0.946993	1.03126	{0.58927, 1.61827}	38.1208	ar	84001.	93018.4	{92887., 93188.}	10.7348
30	R0	{2.88772, 12.4647}	{2.99092, 10.3743}	{{2.99092, 10.3743}, {2.99092, 10.3743}}	{3.57375, 16.7706}	inf	28167.	18655.3	{12797., 26053.}	34.1021	0.196191	newly	14527.	10565.	{6848., 15667.}	31.8885	0.178098	Reff	0.836804	0.464479	{0.295174, 0.661711}	53.7327	ar	93273.	93982.4	{93850., 94113.}	0.760601
31	R0	{2.46831, 8.14308}	{2.98388, 9.60029}	{{2.98388, 9.60029}, {2.98388, 9.60029}}	{20.8876, 17.8951}	inf	22336.	25507.	{20276., 29672.}	21.9452	0.0938574	newly	17282.	16700.4	{12737., 20610.}	14.9287	0.0696157	Reff	0.883087	0.840481	{0.469901, 1.46989}	38.9104	ar	88762.	93948.	{93795., 94112.}	5.84257
32	R0	{1.93236, 7.38987}	{2.9884, 12.8326}	{{2.9884, 12.8326}, {2.9884, 12.8326}}	{54.6503, 73.6512}	inf	13853.	25608.5	{20203., 29739.}	87.2255	0.271663	newly	12032.	12970.3	{9877., 15724.}	17.6406	0.0759391	Reff	0.884615	0.842733	{0.473303, 1.45882}	39.2953	ar	77654.	93983.5	{93851., 94123.}	21.0286
33	R0	{2.82251, 17.6954}	{2.99911, 15.6207}	{{2.99911, 15.6207}, {2.99911, 15.6207}}	{6.25684, 11.7245}	inf	27972.	23834.7	{17045., 28947.}	16.545	0.0886966	newly	10722.	9982.21	{7322., 12729.}	16.8964	0.0820997	Reff	1.00304	0.769109	{0.386345, 1.48348}	49.2585	ar	92745.	94013.6	{93846., 94150.}	1.36789
34	R0	{1.87083, 12.3994}	{2.86095, 19.9929}	{{2.86095, 19.9929}, {2.86095, 19.9929}}	{52.9241, 61.2409}	inf	12842.	23866.8	{18892., 27927.}	88.8462	0.278244	newly	7135.	7996.52	{6247., 9712.}	20.4448	0.0879099	Reff	1.01449	0.820675	{0.470254, 1.24686}	40.3546	ar	76282.	93033.	{92897., 93188.}	21.9593
35	R0	{2.95779, 16.3003}	{2.99911, 15.6207}	{{2.99911, 15.6207}, {2.99911, 15.6207}}	{1.39699, 4.16925}	inf	29124.	23834.7	{17045., 28947.}	18.6299	0.10082	newly	12158.	9982.21	{7322., 12729.}	19.8432	0.104626	Reff	1.01659	0.769109	{0.386345, 1.48348}	49.548	ar	93873.	94013.6	{93846., 94150.}	0.167935
36	R0	{2.18718, 14.261}	{2.8939, 16.8013}	{{2.8939, 16.8013}, {2.8939, 16.8013}}	{32.3119, 17.8129}	inf	18672.	16686.4	{10283., 25758.}	27.5099	0.133968	newly	8858.	6275.62	{3859., 10869.}	36.511	0.207306	Reff	0.915641	0.452107	{0.29376, 0.732301}	55.8493	ar	84379.	93276.2	{93112., 93387.}	10.5444
37	R0	{2.44912, 9.31118}	{2.93597, 11.6547}	{{2.93597, 11.6547}, {2.93597, 11.6547}}	{19.8786, 25.1689}	inf	21427.	25485.2	{20759., 29273.}	23.7997	0.0962205	newly	14344.	14399.7	{10938., 16902.}	13.8769	0.0627396	Reff	0.796601	0.926305	{0.50393, 1.85386}	41.3615	ar	88442.	93584.1	{93441., 93733.}	5.81405
38	R0	{2.05949, 5.72514}	{2.93597, 11.6547}	{{2.93597, 11.6547}, {2.93597, 11.6547}}	{42.5581, 103.571}	inf	16011.	25485.2	{20759., 29273.}	62.1387	0.210062	newly	16782.	14399.7	{10938., 16902.}	14.7022	0.0752636	Reff	0.827297	0.926305	{0.50393, 1.85386}	39.8689	ar	81587.	93584.1	{93441., 93733.}	14.7046
39	R0	{1.75505, 8.52404}	{2.96978, 18.8857}	{{2.96978, 18.8857}, {2.96978, 18.8857}}	{69.2134, 121.558}	inf	10952.	25081.1	{16193., 29530.}	137.183	0.381105	newly	8590.	9197.35	{7326., 10857.}	21.7165	0.109164	Reff	0.957748	1.14561	{0.555705, 2.1851}	46.77	ar	70925.	93825.4	{93652., 93970.}	32.2882
40	R0	{2.24724, 13.7861}	{2.76203, 15.0558}	{{2.76203, 15.0558}, {2.76203, 15.0558}}	{22.9077, 9.21}	inf	18516.	19182.7	{12835., 25593.}	21.311	0.0923571	newly	8935.	8202.83	{5074., 11358.}	23.1479	0.113512	Reff	0.839861	0.632102	{0.354396, 1.10122}	46.0182	ar	85257.	92152.	{91982., 92321.}	8.08733
41	R0	{1.77635, 8.62406}	{2.96947, 15.963}	{{2.96947, 15.963}, {2.96947, 15.963}}	{67.1669, 85.0984}	inf	11281.	24785.9	{19741., 29422.}	123.479	0.350693	newly	8585.	10271.4	{7855., 12460.}	26.8745	0.110245	Reff	0.87725	0.869326	{0.442065, 1.74599}	42.5971	ar	72941.	93829.	{93704., 93974.}	28.6368
42	R0	{1.80397, 5.4932}	{2.93597, 11.6547}	{{2.93597, 11.6547}, {2.93597, 11.6547}}	{62.7505, 112.166}	inf	10692.	21998.7	{16115., 28225.}	105.75	0.304102	newly	11817.	11669.7	{7885., 15980.}	20.7303	0.093433	Reff	0.8332	0.605896	{0.358042, 1.1738}	44.3654	ar	73232.	93584.1	{93441., 93733.}	27.7912
43	R0	{2.83096, 16.7077}	{2.93978, 19.9817}	{{2.93978, 19.9817}, {2.93978, 19.9817}}	{3.84393, 19.5958}	inf	27789.	25201.7	{16725., 29226.}	12.661	0.0934509	newly	11209.	8775.13	{7077., 10205.}	21.7135	0.13395	Reff	0.932207	1.1584	{0.625526, 2.10659}	46.2817	ar	92740.	93627.3	{93451., 93834.}	0.956738
44	R0	{2.73678, 11.6021}	{2.93597, 11.6547}	{{2.93597, 11.6547}, {2.93597, 11.6547}}	{7.27826, 0.453366}	inf	23763.	16775.6	{10741., 26793.}	33.3649	0.188576	newly	12451.	8541.05	{5202., 14396.}	37.3349	0.214047	Reff	0.638655	0.416628	{0.282939, 0.717757}	43.983	ar	92003.	93584.1	{93441., 93733.}	1.71849
45	R0	{2.14235, 6.98601}	{2.99939, 13.7076}	{{2.99939, 13.7076}, {2.99939, 13.7076}}	{40.0047, 96.215}	inf	15339.	25166.	{20084., 29359.}	66.7921	0.221983	newly	13010.	11899.3	{9024., 14406.}	14.6937	0.0730806	Reff	0.718266	0.784675	{0.428583, 1.39244}	40.1426	ar	82974.	94036.2	{93909., 94200.}	13.3322
46	R0	{2.23409, 9.21289}	{2.86001, 13.5542}	{{2.86001, 13.5542}, {2.86001, 13.5542}}	{28.0168, 47.1221}	inf	19256.	24182.	{20636., 28065.}	35.6104	0.148989	newly	14108.	12127.1	{9217., 14418.}	14.8288	0.0837021	Reff	0.983357	1.0564	{0.545261, 1.73337}	41.7665	ar	85163.	92998.7	{92780., 93186.}	9.20079
47	R0	{2.12258, 8.15265}	{2.93597, 11.6547}	{{2.93597, 11.6547}, {2.93597, 11.6547}}	{38.3208, 42.956}	inf	17054.	25485.2	{20759., 29273.}	52.6236	0.184452	newly	13477.	14399.7	{10938., 16902.}	16.3577	0.0694407	Reff	0.871829	0.926305	{0.50393, 1.85386}	38.3134	ar	82690.	93584.1	{93441., 93733.}	13.1746
48	R0	{2.71771, 10.4636}	{2.93597, 11.6547}	{{2.93597, 11.6547}, {2.93597, 11.6547}}	{8.03103, 11.3833}	inf	26340.	25485.2	{20759., 29273.}	11.9266	0.0595296	newly	16548.	14399.7	{10938., 16902.}	14.0554	0.0715782	Reff	0.927228	0.926305	{0.50393, 1.85386}	37.744	ar	91716.	93584.1	{93441., 93733.}	2.03679
49	R0	{2.44369, 6.66477}	{2.9751, 8.95585}	{{2.9751, 8.95585}, {2.9751, 8.95585}}	{21.7462, 34.376}	inf	18124.	22291.5	{16956., 28593.}	26.9644	0.0995381	newly	14957.	14780.2	{10336., 20202.}	21.8657	0.0977536	Reff	0.592717	0.616804	{0.356298, 0.877149}	40.5992	ar	88827.	93878.8	{93754., 94012.}	5.68721
50	R0	{2.77383, 17.9775}	{2.96978, 18.8857}	{{2.96978, 18.8857}, {2.96978, 18.8857}}	{7.06424, 5.05187}	inf	25584.	24267.7	{17651., 29319.}	15.4961	0.0846424	newly	9271.	8627.73	{6319., 10754.}	15.576	0.080485	Reff	0.747852	0.89663	{0.432192, 1.75752}	50.8963	ar	92334.	93825.4	{93652., 93970.}	1.61523
